# Electric charging/discharging characteristics of super capacitor, using de-alloying and anodic oxidized Ti-Ni-Si amorphous alloy ribbons

**DOI:** 10.1186/1556-276X-9-253

**Published:** 2014-05-21

**Authors:** Mikio Fukuhara, Kazuyuki Sugawara

**Affiliations:** 1 New Industry Creation Hatchery Center, Tohoku University, 3-4-1, Sakuragi, Tagajyo, Miyagi 985-8589, Japan; 2 Fracture and Reliability Research Institute, Tohoku University, Sendai 980-8579, Japan; 3 Green Device Laboratory, Institute for Nanoscience & Nanotechnology, Waseda University, Shinjuku, Tokyo 162-0041, Japan; 4 Research Institute for Electromagnetic Materials, Sendai, Miyagi 982-0807, Japan

**Keywords:** Electric storage devices, Amorphous alloys with TiO_2-x_ surfaces, Nanometer sized cavity

## Abstract

Charging/discharging behaviors of de-alloyed and anodic oxidized Ti-Ni-Si amorphous alloy ribbons were measured as a function of current between 10 pA and 100 mA, using galvanostatic charge/discharging method. In sharp contrast to conventional electric double layer capacitor (EDLC), discharging behaviors for voltage under constant currents of 1, 10 and 100 mA after 1.8 ks charging at 100 mA show parabolic decrease, demonstrating direct electric storage without solvents. The supercapacitors, devices that store electric charge on their amorphous TiO_2-x_ surfaces that contain many 70-nm sized cavities, show the Ragone plot which locates at lower energy density region near the 2nd cells, and *RC* constant of 800 s (at 1 mHz), which is 157,000 times larger than that (5 ms) in EDLC.

## Introduction

Titanium oxide (TiO_2_) is of considerable interest for wide range of applications, including photocatalysis [1], optovoltaics [2], solar energy conversion [3], chemical sensors [4], bioprobes [5] and environmental pollution control [6]. Although the majority of the applications of TiO_2_ are generally controlled by the crystalline phase [7], we report distinguished amorphous material supercapacitors, devices that store electric charge on their amorphous titanium oxide surfaces that contain many 70-nm sized cavities.

Following the capacitance studies of Ni-Nb-Zr-H glassy alloys with femtofarad capacitance tunnels [8,9], we have found that the capacitance of nanocrystalline de-alloyed Si-Al [10,11] or Si-Al-V [12], and de-alloyed and anodic oxidised amorphous Ti-Ni-Si alloy ribbons [13] show prompt charging/discharging of 102 μF (0.55 F/cm^3^) at a frequency of 1 mHz, from 193 to 453 K, and with a high voltage variation from 10 to 150 V. Especially, the de-alloyed and anodic oxidized Ti-Ni-Si alloy one displayed a capacitance of ~ 4.8 F (~52 kF/cm^3^) in discharging behaviors for voltage after 1.8 ks charging at DC current of 100 mA [13]. We assume that the surface structure of the oxide consists of a distributed constant equivalent circuit of resistance and capacitance, analogous to active carbons in electric double-layer capacitors (EDLCs). The amorphous materials of interest are completely different from the conventional “wet” cells such as EDLC and secondary cells which are controlled by diffusivity of ions. We termed this device a “dry” electric distributed constant capacitor (EDCC).

In this study, we report DC and AC charging/discharging characteristics for anodic oxidized-amorphous Ti-15 at.% Ni-15 at.% Si alloy supercapacitors with higher narrow cavity densities and higher electric resistivities, with an aim to obtain further wide behaviors for Ti-Ni-Si ones, in comparison with those of the de-alloyed Si-Al alloy one [10,11].

## Experimental

### Materials

The rotating wheel method under an He atmosphere was used for preparing Ti-15 at.% Ni-15 at.% Si alloy ribbons of 1 mm width and a thickness of about 50 μm, using a single-wheel melt-quenching apparatus (NEV-A05-R10S, Nisshin Gikken, Saitama, Japan) with rotating speed of 52.3 m/s. De-alloying and anodic oxidation of the specimens were carried out for 288 ks in 1 N HCl solution and for 3.6 ks in 0.5 Mol H_2_SO_4_ solution at 50 V and 278 K, respectively. The densities of the specimen before and after surface treatment were 4.424 and 3.878 Mg/cm^3^, respectively.

### Characterization

The phase transformations upon heating were studied by differential scanning calorimetry (DSC) at a heating rate of 0.31 K/s using 10-mg specimens. The structure of specimens was identified by X-ray diffraction with Cu Kα radiation in the grazing incidence mode. Topography images were observed using a noncontact atomic force microscope (NC-AFM, JSPM-5200, JEOL, Akishima, Tokyo, Japan). A scanning Kelvin probe force microscopy (SKPM) based on the measurement of electrostatic force gradient was applied to measure an absolute electrical potential between the cantilever tip coated with Pt at 0 eV and TiO_2_ surface as the work function difference.

### Discharging measurement

The specimen (1 mm wide, 50 μm thick, and 10 mm long) with double–oxidized surface was sandwiched directly by two copper ribbons beneath two pieces of glass plates using a clamp. Capacitances were calculated as a function of frequency between 1 mHz and 1 MHz from AC electric charge/discharge pulse curves of 10 V applied at 25 ns ~ 0.1 s intervals, using a mixed-signal oscilloscope (MSO 5104, Tektronix, Beaverton, OR, USA) and 30 MHz multifunction generator (WF1973, NF Co, Yokohama, Japan) on the basis of a simple exponential transient analysis. The charging/discharging behavior of the specimen was analyzed using galvanostatic charge/discharge on a potentiostat/galvanostat (SP-150, BioLogic Science Instruments, Claix, France) with DC’s of 10 V, 10 pA ~ 100 mA for ~900 s at room temperature. The details of the procedure have been described in previous paper [13]. Experimental inspection for electric storage was carried out by swing of reflected light of DC Galvanometer (G-3A, Yokogawa Electric, Tokyo, Japan) after charging at 1 mA for 20 s.

## Results and discussion

### Thermoanalysis and phase analysis of anodic oxidized alloys

**Figure 1:**
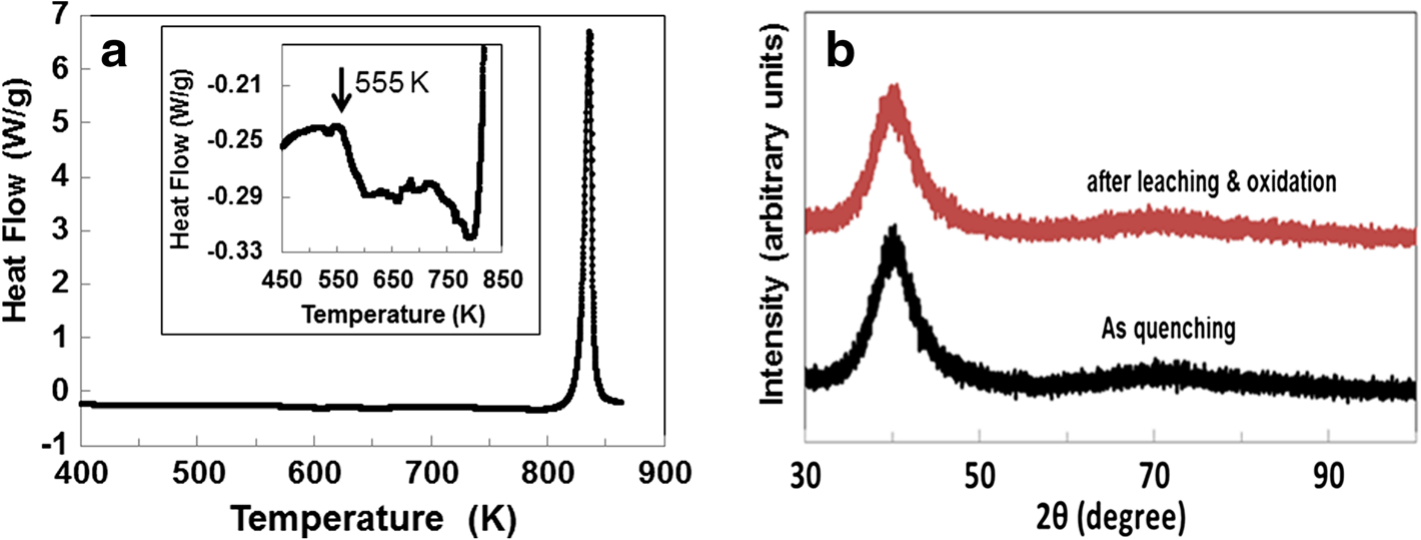
**DSC trace and X-ray diffraction patterns.** DSC trace of the studied amorphous Ti-Ni-Si alloy scanned at 0.67 K/s **(a)** and X-ray diffraction patterns of the studied alloy before and after de-alloying and then anodic oxidation **(b)**.

The DSC trace of the studied Ti-15 at% Ni-15 at% Si alloy ribbons shown in Figure 1a exhibits an increment in *Cp* at the glass transition temperature (*Tg*) of 555 K and one clear exothermal peak with peak temperature of 836 K. Referring to the DSC trace (Figure 1a), Figure 1b shows X-ray patterns of the specimen before and after de-alloying and anodic oxidization. It is known that amorphous titanium oxide exists in nonstoichiometric form, TiO_2-x_ which has a complicated defect structure [14].

### Morphological and dielectric analysis of anodic oxidized alloys

**Figure 2:**
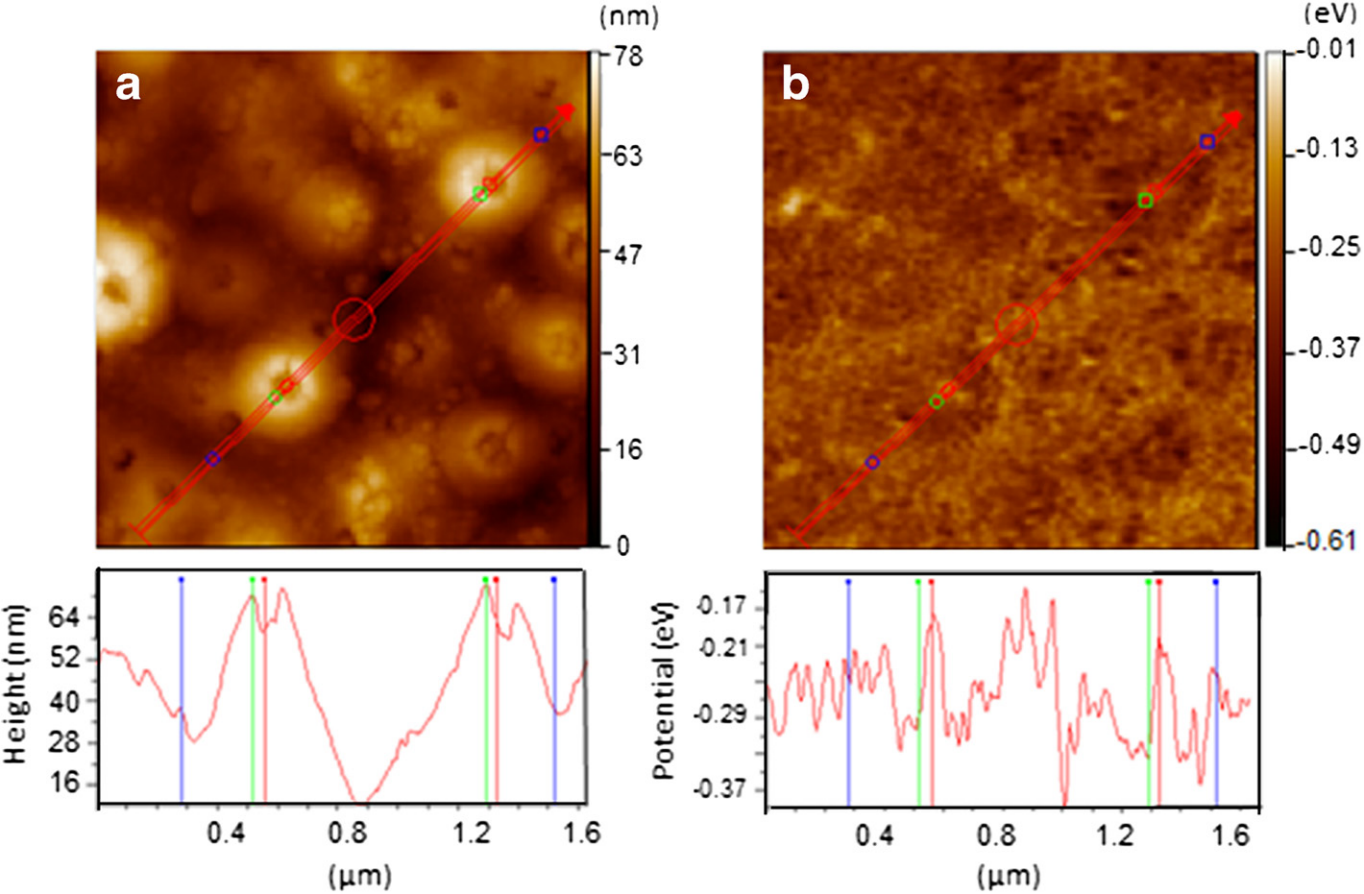
**AFM image (a) and corresponding SKPM image (b) for surface of de-alloyed and then anodic oxidized Ti-Ni-Si specimen.** Lower profiles of **(a)** and **(b)** are height from valley bottom and electrostatic potential for probe with 0 eV along red lines in upper images, respectively.

Figure 2a and b show the atomic force microscope (AFM) images and the corresponding scanning Kelvin probe force microscope (SKPM) images for oxidized speccimens, respectively. The image in Figure 2a shows that a large numbers of volcanic craters with round pores approximately 70 nm in diameter were formed on the titanium oxide surface [15,16]. The profile line length of Figure 2a shows 2.5 times longer than smooth one defore anodic oxidation, indicating increment of the surface area by around 6 times. From the line profiles of the noncontact AFM (NC-AFM), spots ca. 7 nm in size with higher work functions *Φ*, of 5.53 eV (=5.65 (*Φ*_*Pt*_)–0.12 (*Φ*_*CPD*_)) are located in volcanic craters and at the bottom of ravines. The concave contact potential difference *Φ*_*CPD*_, indicates storage of electric charges [17].

### DC charging/discharging activity of EDCC

**Figure 3:**
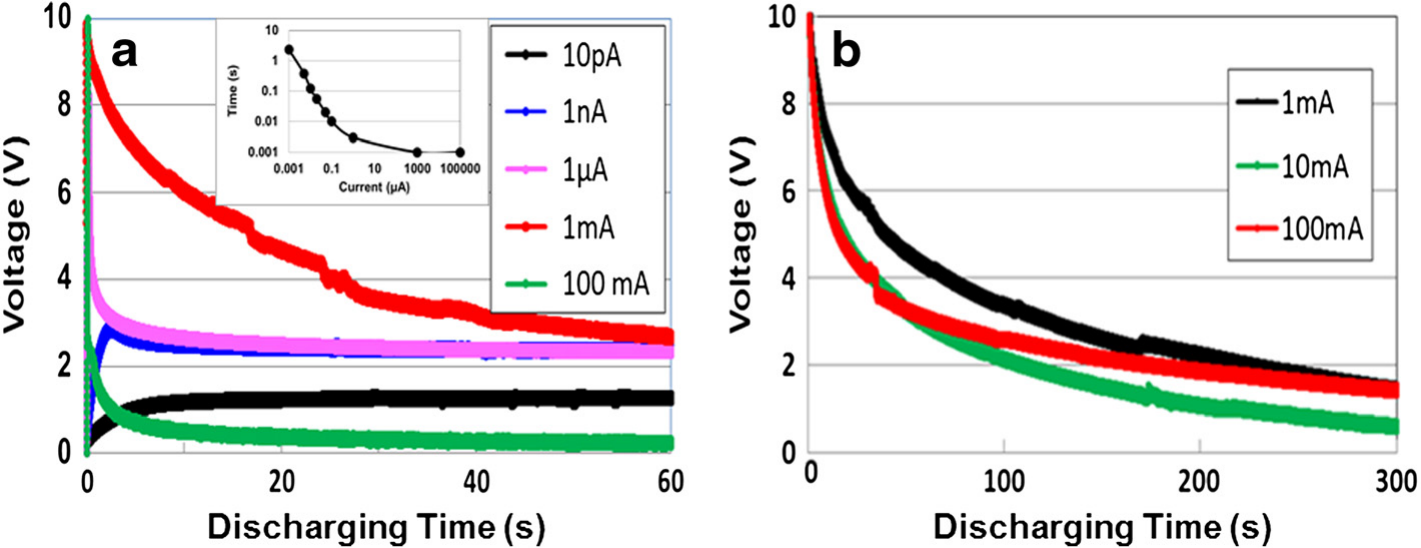
**Self-discharge curves and discharging behaviors. (a)** Self-discharge curves after charging at current of 10 pA, 1 nA, 1 μA, 1 mA, and 100 mA for approximately 0.5 s. The inset shows the current effect on the charging time up to 10 V. **(b)** Discharging behaviors for voltage under constant currents of 1 mA, 10 mA, and 100 mA after 1.8-ks charging at 100 mA.

**Figure 4:**
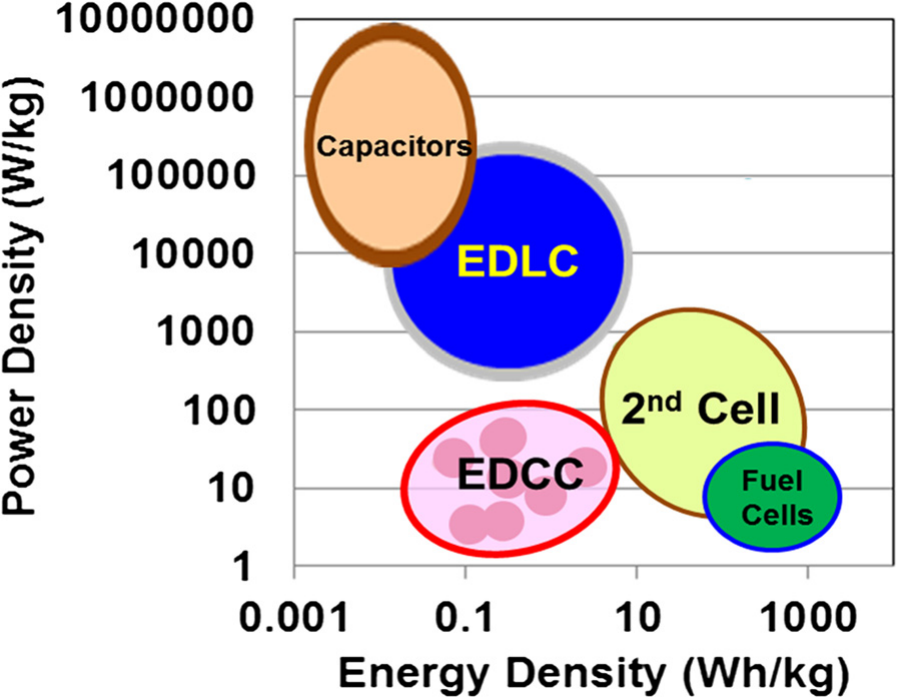
**Comparison of the power density and energy density.** For EDCC, EDLC, batteries, and fuel cells in Ragon plot (after Whittingham [20]).

The self-discharge curves of the EDCC device after charging at DC currents of 10 pA ~ 100 mA for ~ 0.5 s are shown in Figure 3a, along with the current effect on charging-up time. Lower current of 1 nA cannot reach 10 V, but current increments reduce charging time up to 10 V (inset). We see an ohmic *IR* drop after charging at above 1 μA, which is characteristic of EDLCs [18]. The three curves at or above currents of 1 μA decrease parabolically after charging, indicating internal charging of unsaturated cells (the potential drop caused by current passing through resistive elements in an equipment circuit of the matrix [19]). Therefore, a long discharge time is necessary to charge completely the large number of capacitor cells in the EDCCs as well as the EDLCs [18,19]. Since a charge of 100 mA suppresses the voltage decrease in the discharging run, we then measured the discharging behavior under constant current of 1, 10 and 100 mA after 1.8 ks of charging at 100 mA. These results are presented in Figure 3b. From straight lines in curves, we obtained a capacitance *C* of ~17 mF (~8.7 F/cm^3^), using formulae of power density *P* and energy density *E*, *P* = *IV*/kg and *E* = *P*Δ*t*, respectively, where Δ*t* is the discharge time. The Ragone plot, the relation between energy density and power density, is presented in Figure 4, along with conventional capacitors, EDLC, the 2nd and fuel cells [20]. The plot is located at lower energy density region near the 2nd cells. It needs further improvement for energy density.

### AC electric measurement of EDCC

**Figure 5:**
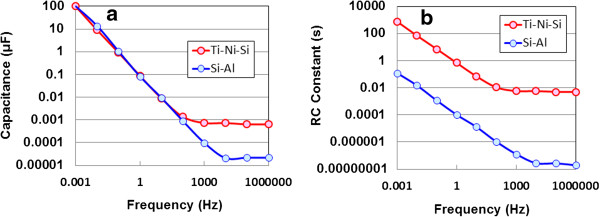
**Frequency dependence of capacitance (a) and RC constant (b).** For de-alloyed and anodic oxidized Ti-Ni-Si and de-alloyed Si-Al specimens in an input voltage of 10 V at room temperature.

Capacitance as a function of frequency at room temperature is presented logarithmically in Figure 5a, along with those of the de-alloyed Si-20at%Al specimen [11]. Frequency dependent capacitances decreased parabolic from around 0.1 mF (0.54 F/cm^3^) to around 1.3 pF (53 μF/cm^3^) with increasing frequency and saturated from 0.1 to 0.4 nF in frequency region from 1 kHz to 1 MHz. The saturated values of the former are 30 times larger than those of the latter. This difference would be derived from higher absorbed electron density of the former, accessible to electron trapping. Here it should be noted that charging/discharging of electrochemical cells occurs at lower frequency regions on the whole interfaces in pores of electrodes, but does not occur at higher frequency ones in interior parts of pores [21]. Hence, by analogy we infer that that the de-alloyed and anodic oxidized Ti-Ni-Si material, which shows large frequency dependence on capacitance independent of temperature, is an assembly of canyons with the deepest recess. The whole behavior in Figure 5a implies ac current momentary (below 0.1 s) charging/discharging, with the observed decrease in capacitance come from dielectric dispersion by interfacial polarization. These results would be associated with electron storage in amorphous TiO_2-x_ coated solid cell without solvents. Furthermore, we can store electricity in ac current using a rectifier, if we could be taken a figure up three places over capacitance at higher frequencies.

Figure 5b shows a frequency dependent RC constant in input voltage of 10 V at room temperature for the former and the latter [11]. The former’s RC decreases parabolically from around 800 s (13.1 min) to around 5 ms with increasing frequency up to 1 kHz at 100 ms-15 ns intervals, before becoming saturated in the frequency region from 1 kHz to 1 MHz. The 800 s (13.1 min) at 1 mHz is 157,000 times larger than that (5 ms) in the conventional EDLC [19]. However, it needs larger ones from 0.1 s to few hours for practical use. On the other hand, the latter’s RC constants are 4–5 times smaller than those of the former in whole frequency region.

### Electric storage inspection of EDCC

**Figure 6:**
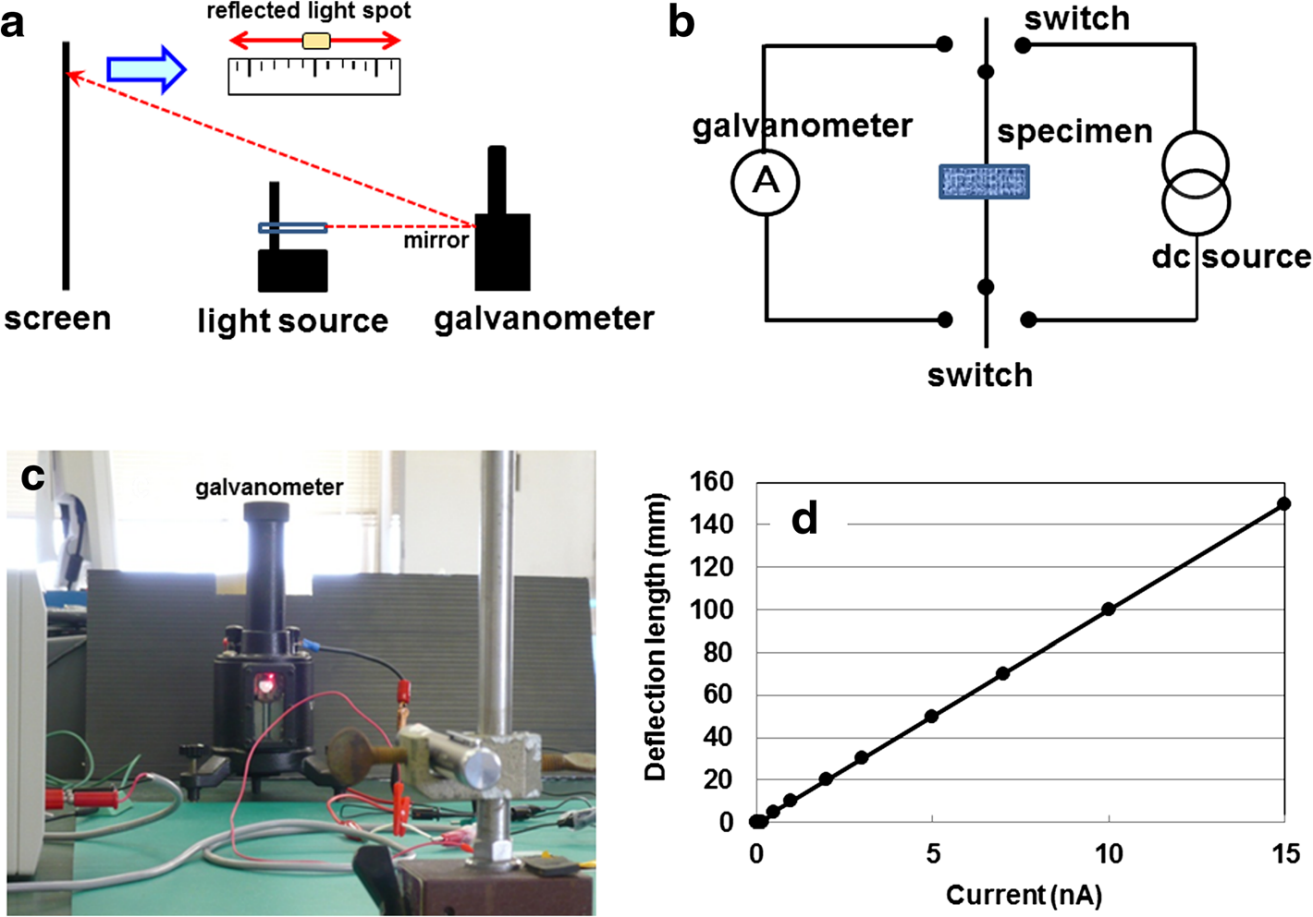
**Experimental inspection figures for electric storage by swing of reflected light of galvanometer. (a)** Schematic experimental view, **(b)** experimental circuit, **(c)** experimental view, and **(d)** relation between deflection length on screen and current for this system.

To provide visible proof for electric storage of the EDCC, we observed a swing of reflected light of galvanometer with mirror on a rotating magnetic ring. The schematic experimental system is presented in Figure 6, which is composed of schematic experimental view (a), experimental circuit (b), experimental view (c), and calibration line between deflection length on screen and current for this system (d). In Additional file 1: Movie 1, the reflected light spot begins to swing slowly from right to left, then gradually slows down, and lastly stops at seven rounds of around 60 s due to complete consumption of the electric power, charged at 1 mA for 20 s.

## Conclusion

Amorphous Ti-15 at.% Ni-15 at.% Si alloys prepared by the rotating wheel method were leached out for 288 ks in 1 N HCl solution at room temperature and anodically oxized for 3.6 ks in 0.5 M H_2_SO_4_ solution at 50 V and 278 K, respectively. AFM images showed a large numbers of volcanic craters with round pores approximately 70 nm in diameter on amorphous TiO_2-x_ surface. The line profiles of the NC-AFM revealed spots ca. 7 nm in size with higher work functions of 5.53 eV in volcanic craters and at the bottom of ravines, indicating storage of electric charges.

DC discharging behaviors of the EDCC devices for voltage under constant currents of 1, 10 and 100 mA after 1.8 ks charging at 100 mA show parabolic decrease, demonstrating direct electric storage without solvents. In comparison of the power density and energy density for EDCC, the Ragone plot is hardly much for the 2nd cells. In sharp contrast to the de-alloyed Si-20at%Al specimen, frequency dependent capacitance and RC constant in input voltage of 10 V at room temperature for the Ti based one show 30 times larger in frequency region from 1 kHz to 1 MHz and 4–5 times larger in whole frequency region, respectively. The 800 s of the Ti based one at 1 mHz is 157,000 times larger than that (5 ms) in the conventional EDLC, lying in practical use region from 0.1 s to few hours. The 65 s-swing of reflected light spot in Movie clearly demonstrates electric storage of EDCC used in this study.

## Competing interests

The authors declare that they have no competing interests.

## Authors’ contributions

FM conceived the idea of de-alloying and anodic oxidized supercapacitor, designed the amorphous materials, measured charging/discharging behaviors, and wrote the manuscript. SK participated in fabrication of devices and performed their characterizations. Both authors read and approved the final manuscript.

## Supplementary Material

Additional file 1 **Movie 1.** The swinging movie of the reflected light spot at seven rounds for around 60 s as an evidence of the electric power charged. Click here for file
